# Can evidence drive health equity in the COVID-19 pandemic and beyond?

**DOI:** 10.1057/s41271-023-00452-3

**Published:** 2024-01-12

**Authors:** Katy Bell, Sam White, Abbey Diaz, Priya Bahria, Fiona Sima, Wael K. Al-Delaimy, Susan dosReis, Omar Hassan, Dorothy Drabarek, Monjura Nisha, Kesha Baptiste-Roberts, Katy Gwiazdon, Camille Raynes-Greenow, Robin Taylor Wilson, James A. Gaudino, Rafael da Silveira Moreira, Bruce Jennings, Pauline Gulliver

**Affiliations:** 1 Sydney School of Public Health, Sydney Medical School, Faculty of Medicine and Health, The University of Sydney, Edward Ford Building (A27), Camperdown, Sydney, NSW 2006 Australia; 2 International Network for Epidemiology in Policy, Sydney, NSW Australia; 3 First Nations Cancer and Wellbeing Research Team, School of Public Health, University of Queensland, Brisbane, QLD Australia; 4 European Medicines Agency, Amsterdam, The Netherlands; 5 Institute for Health Research, University of Bedfordshire, Luton, England, UK; 6 Herbert Wertheim School of Public Health and Human Longevity Science, University of California San Diego, San Diego, CA USA; 7 University of Maryland School of Pharmacy, Pharmaceutical Health Services Research, Baltimore, MD USA; 8 Department of Public Health Analysis, School of Community Health and Policy, Morgan State University, Baltimore, MD USA; 9 Center for Environmental Ethics and Law, Vienna, VA USA; 10 Department of Epidemiology & Biostatistics, College of Public Health, Temple University, Philadelphia, PA USA; 11 School of Public Health, Oregon Health & Sciences University and Portland State University, Portland, OR USA; 12 Area of Social Medicine, Faculty of Medicine, Federal University of Pernambuco, Recife, Pernambuco Brazil; 13 Center for Biomedical Ethics and Society, Vanderbilt University, Nashville, TN USA; 14 Section of Social and Community Health, University of Auckland, Auckland, New Zealand

**Keywords:** COVID-19, Health equity, Heath policy, Social determinants of health, Epidemiology

## Abstract

Using scoping review methods, we systematically searched multiple online databases for publications in the first year of the pandemic that proposed pragmatic population or health system-level solutions to health inequities. We found 77 publications with proposed solutions to pandemic-related health inequities. Most were commentaries, letters, or editorials from the USA, offering untested solutions, and no robust evidence on effectiveness. Some of the proposed solutions could unintentionally exacerbate health inequities. We call on health policymakers to co-create, co-design, and co-produce equity-focussed, evidence-based interventions with communities, focussing on those most at risk to protect the population as a whole. Epidemiologists collaborating with people from other relevant disciplines may provide methodological expertise for these processes. As epidemiologists, we must interrogate our own methods to avoid propagating any unscientific biases we may hold. Epidemiology must be used to address, and never exacerbate, health inequities—in the pandemic and beyond.

**Supplementary Information:**

The online version contains supplementary material available at 10.1057/s41271-023-00452-3.

## Key messages

- We call on health policymakers to co-create, co-design, and co-produce equity-focussed, evidence-based interventions with communities, focussing on those most at risk to protect the population as a whole.
- Epidemiologists and other Public Health researchers must interrogate our own methods to avoid propagating any unscientific biases we may hold.
- Epidemiology must be used to address, and never exacerbate, health inequities

## Introduction

Throughout the COVID-19 pandemic, a recurring narrative of politicians and media globally indicated that the SARS-CoV-2 virus ‘does not discriminate’. But risks and vulnerabilities experienced by communities have not been equally distributed [1]. There have been substantial health inequities observed among different groups. Within and across countries researchers have reported unequal risk of infection, access to testing, access to treatment, short- and long-term morbidity, and mortality [2–7].

Braveman [8] and Marmot [9] defined health inequities as unfair and avoidable systematic discrepancies in the opportunities different groups have to achieve positive health outcomes. Inequities differ from health inequalities, which describe differences in health outcomes between groups or individuals (irrespective of opportunities to avoid adverse outcomes) [10]. In the UK and some other countries, people intend the same meaning with both words and use them interchangeably [9, 11]. Amplification of existing health inequities causing further inequalities in health outcomes during pandemics is not new. A higher incidence of infection among the American working class during the 1918 influenza pandemic [12] challenged the widely held consensus that ‘the flu hit the rich and the poor alike’. Similarly, the mortality rate among Māori during the 1918 influenza pandemic reached 7.3 times that of non-Māori in the Aotearoa New Zealand population [13]. Even during the 2009 H1N1 influenza outbreak, researchers reported considerable discrepancies in mortality between different socio-economic and racially minoritised groups [14–16]. Such examples point to longstanding, complex underlying systems that favour the health and wellbeing of some over others.

Race is a socially constructed classification system used to distinguish between populations with similar phenotypical characteristics [17, 18]. The absence of genetic or biologically based differences between racial groups does not negate the concept of race and negative impacts of racism [18, 19]. Structural racism is not simply the result of prejudices held by individuals, but is produced and reproduced by the laws and practices that underpin how governments, economies, and societies function [20]. Groups with disproportionately poorer health outcomes during the COVID-19 pandemic include racially minoritised groups [17], immigrants and refugees, older people, people with immunocompromise, pregnant people, those with disabilities, in socio-economic poverty, living with home insecurity, or who are incarcerated. Many of these are ‘intersectional’ (occur together) in individuals and among groups, further magnifying inequities [21]. The concept of intersectionality originated with recognition that harm from the violence against women is amplified by the violence of racism [22] among individual experiencing the dual violence. Intersectionality may be understood and applied in diverse ways [23].

Several studies have identified the COVID-19 pandemic as a global challenge that has increased health inequities [2–6]. Of the multiple disciplines involved in health inequities research [24], as representatives of the *International Network for Epidemiology in Policy (INEP)*, we are particularly interested in the role that epidemiology may play. Thus, we undertook this scoping review to systematically search, identify, and collate published, well-described, and policy-relevant approaches in which someone has applied epidemiological methods to COVID-19 pandemic inequities in healthcare and health outcomes. We then critically assessed the potential of proposals for addressing pandemic-related health inequities.

## Methods

We conducted this scoping review in accordance with the Joanna Briggs Institute methodology [25] and report our findings in accord with the Preferred Reporting Items for Systematic Reviews and Meta-Analyses extension for Scoping Reviews (PRISMA-ScR) checklist [26]. We searched 11 databases for relevant articles published from 1 January 2020 through to 17 February 2021 to identify and synthesise published scientific literature describing policy-relevant and evidence-based approaches using epidemiological methods (broadly defined [27]) to address health inequities related to the COVID-19 pandemic. We provide details of all steps of the search and assessment in Supplementary Material Part 1. After collating the evidence and organising it into themes, the authors, all members of the International Network for Epidemiology in Policy Equity working group, considered the proposals and provided commentary. The authors are academics, researchers, health practitioners, and policy experts with diverse gender, country of birth and residence, and ethnic and cultural backgrounds. Although the majority are epidemiologists, one is a lawyer and one is a bioethicist. This diversity was reflected in robust discussion among us about the merits and disadvantages of the proposals. We recognise that our commentary reflects our combined views and experiences and is itself open to critique by others.

## Results

Our search retrieved 2623 unique records for screening of title and abstracts, 243 records with full texts; we excluded 165 of these and included 77 records in our review (see Supplementary Material Part 2). The 77 records (Supplementary Table S1) included 66 commentaries; 3 letters, 2 case reports, 2 policy reviews, an ethical analysis, a modelling study, a description of a framework, and an editorial. Most records focussed on single countries, especially the United States (US) (*n* = 48); 17 had a global focus and 2 regional (Africa and Americas). Many articles (*n* = 50) addressed systematic causes of inequity at the population level, including a focus on racially minoritised groups. Seven articles each focussed on inequities for older people and for people with disabilities; only two focussed on children, and one on pregnant people. In total, 18 of the included studies addressed risk of infection and of morbidity and mortality from COVID-19 (Table S2), 13 addressed access to COVID-19 tests and vaccines (Table S3), 15 addressed access to treatment for COVID-19 (Table S4), 8 addressed non-COVID-19 morbidity and mortality (Table S5), and 23 addressed multiple inequities (Table S6).

### Inequity in risk of infection, morbidity, and mortality from COVID-19

#### Proposals

Authors suggested ways to address inequity in risk of infection including increased provision of personal protective equipment (PPE) and increased capacity for social distancing in underserved communities and settings (Supplementary Material Table S2 and Box S1). Henry et al. recommended implementing rapid release of suitable individuals from incarceration to help ensure US prisons were capable of adhering to social distancing guidelines [28]. Other commentaries discussed prison reform and emphasised preventing incarceration. Recommendations to reduce the inequitable risk of infection for older people, and to strengthen the aged care workforce, include improved pay and paid sick leave to obviate need for carers to work multiple jobs (increasing their own risks of infection). Adebisi et al. recommended decriminalisation of sex work in Africa [29]. Bonn et al. advocated for moratoria on enforcement of laws criminalising illicit drug use [30]. Several authors recommended moratoria on housing evictions to reduce community transmission of COVID-19 in racially minoritised groups at increased risk of eviction. To improve transparency of reporting, some suggested collection and release of COVID-19 data by (self-reported) race, ethnicity, and age so that resources could be targeted to populations in need.

#### INEP commentary

Approaches need to address inequities for risk of adverse outcomes from infection, as well as inequities in risk of infection. Proposals primarily targeted individuals and neglected structural systems of disadvantage which place entire communities at increased risk. Communities may have intergenerational households without space to physically distance or may lack access to clean water for hand hygiene. Historical considerations contribute to inequities such as ‘red-lining’ in the US—a government practice that placed Black communities in undesirable areas close to toxic industries, major thoroughfares (with increased air pollution), or without proper infrastructure (including access to adequate food, water, and healthcare). Many still live in those same areas and have experienced generations of public health disadvantages that increased their susceptibility to harmful pathogens.

### Inequity in access to testing and to vaccines for COVID-19

#### Proposals

To increase equitable access to testing and vaccines against COVID-19 researchers suggested mobile COVID-19 testing and vaccination centres in popular community spaces such as pharmacies, physician offices, churches, and schools in communities (Table S3 and Box S2). These were proposed to benefit Aboriginal and Torres Strait Islander communities in Australia and Black American communities in the US. Other suggestions included developing equity-based allocation frameworks and including underrepresented groups in vaccine clinical trials.

Some researchers discussed the impact of economic inequity between high- and low-income countries on vaccine allocation. They discussed models for equity-based global vaccine allocation including the Covax proportional allocation model and the Fair Priority model [31]. Abbas proposed tiered pricing of vaccines according to the purchasing power of countries. After negotiation with the government of Brazil, GlaxoSmithKline (GSK) agreed to sell its 10-valent vaccine for $7 per dose although the firm was selling at $56 and $71 per dose in Europe and the US, respectively. Herzog acknowledged that a system of allocating vaccines according to the population of different countries would provide a fairer and more efficient system compared to an open market [31]. Herzog contended, however, that this could be improved on by adopting the Fair Priority model, a 3-phase system, that also factors in metrics such as standard expected years of life lost averted per dose and loss of gross national income. Herzog argued that this model more closely aligns with the World Health Organization (WHO) values of beneficence, equal moral concern, and prioritising the underserved.

A key feature of several models was early allocation of vaccines to older people to minimise mortality. Additional recommendations included the use of population-based randomised trials for roll-out of population vaccination programs, and countering vaccine hesitancy due to misinformation and mistrust of the healthcare system. For improving communication, researchers emphasised the importance of culturally and linguistically diverse, evidence-based public health messages and engaging with social influencers and leaders of cultural and faith-based groups to send them.

#### INEP commentary

Most of the included papers focussed on equitable vaccine allocation within countries, likely reflecting immediate priorities and available policy levers. COVID-19 vaccine access and equity, however, is a global problem, with refugees and undocumented migrants among the most vulnerable [32]. Although some records (highlighted above) discussed inequity between higher and lower income countries, this was not their primary focus and none mentioned potential ways to address to this urgent problem. These include (1) patent waivers to permit local vaccine production, (2) use of the World Trade Organisation Trade-Related Aspects of Intellectual Property Right (TRIPS) agreement to allow production of vaccines outside patent protection, and (3) sales of vaccines at cost price (to the pharmaceutical company) to eligible countries (such as the United Nations–backed Medicines Patent Pool) [33].

Within countries, use of age-based prioritisation for vaccine distribution may have been overly simplistic and missed the intersectional nature of morbidity and mortality from COVID-19. Vaccine roll-out programs run in partnership with communities tended to be more successful. In the US, Indigenous American groups given responsibility to vaccinate in their communities had higher rates than the rest of the US [34]. Local ownership of testing and vaccination messaging and facilities could also be helpful in historically disadvantaged communities, such as those noted.

Although most solutions aimed to increase vaccine uptake by improving the clarity and relevance of messaging to communities, we need research to identify other potential barriers to COVID-19 vaccination that may be redressed. Messaging may need to acknowledge mistrust from systemic racism and historical injustice before providing information about the vaccines [35]. Another key barrier noted is out-of-pocket costs associated with vaccination, particularly in countries without universal healthcare coverage.

### Inequity in access to treatment for COVID-19

#### Proposals

Several records discussed the importance of providing linguistically and culturally tailored medical care to individuals infected with COVID-19 (Table S4, Box S3). One recommendation is to deploy clinicians fluent in the preferred community language. A hospital in Massachusetts, US, implemented this initiative with 51 bilingual physicians representing 14 countries of origin who provided 14-h coverage in support to the medical team [36]. Some, including Gill et al. [37], suggested including underrepresented racially minoritised groups and people with sociodemographic disadvantages in phase 3 clinical trials as a way to increase access to, and the evidence base for treatments for these population groups.

Essien et al. [38] suggested increasing diversity among hospital triage committees and revising critical care triage guidelines to prevent underserved groups from experiencing discriminatory medical care. White and Lo [39] suggested existing triage guideline could use correction factors to reduce the impact of structural inequities, prioritise high-risk essential workers, and reject longer-term survival as an allocation criterion. Schmidt et al. [40] criticised the Sequential Organ Failure Assessment algorithm’s use of a single ‘colour-blind’ serum creatinine threshold to estimate a patient’s probability of dying in the Intensive Care Unit. This practice systematically discriminates against Black Americans who tend to have higher serum creatinine levels [40]. Erasmus [41] recommended aligning South African Intensive Care Unit triage guidelines with the South African Constitution to formally protect older people and persons with disabilities from discrimination. Brown and Goodwin [42] raised the importance of limiting consideration of disabilities and chronic illnesses that do not affect prognosis in COVID-19 infection in Intensive Care Unit triage criteria.

#### INEP commentary

Despite a suggestion of increasing representation in trials, no author explored ways to achieve this. Partnership with communities for the co-creation, co-design, and co-production of research projects and interventions may ensure the trial is fit for purpose and enhance the translation of findings into equitable practice changes [43]. Culturally safe recruitment strategies are also likely to be important. Some of the proposals could worsen inequities faced by older people already disproportionately affected by COVID-19 [44]. Proposals for addressing access to treatment for those with severe disease did not consider that many COVID-19 treatment algorithms inherently discriminate against older people due to in-built utilitarian biases favouring individuals with greater ‘future productivity’. This bias may be amplified in people from minoritised groups, where “weathering” from the effects of sustained cultural oppression means these individuals have become biologically older than their chronological age [45]. Policy makers and practitioners need to examine algorithms for potential discrimination from in-built biases in the data or decisions made in their development.

### Non-COVID-19 morbidity and mortality

#### Proposals

Several records promoted the uptake of telemedicine and patient care options that limit face-to-face interactions to assist in reducing inequities relating to non-COVID-19 morbidity and mortality (Table S5, Box S4). Proposals in the US included Congressional allocation of funding from the COVID-19 telehealth program to community clinics and action by the Federal Communication Commission to incentivise making broadband internet access more equitably available. Valdez et al. [46] emphasised the importance of developing accessible telemedicine approaches to optimise the participation of persons with disabilities. This is particularly important in rural communities where remoteness and disability can be intersectional and thereby compound difficulty of accessing internet-based services. Valdez et al. [46] recommended developing accessible telemedicine software to ensure compatibility with external assistive technology devices, incorporating plug-ins that allow for sign language or closed captioning, making user-friendly interfaces that use both icons and text, and enabling multiple users of the same account to incorporate others in consultations by proxy. Only two records [47, 48] mentioned disruption to children’s education and subsequent impacts on their social, economic, and health needs due to school closures. Armitage et al. [48] advocated replacing school closures with strategies to reduce COVID-19 transmission among students (smaller class sizes, physical distancing in classrooms and promotion of good hygiene practices.)

#### INEP commentary

Although telemedicine has the potential to facilitate physically distanced healthcare interactions, it may exclude people without reliable access to internet or electronic devices who may experience worsening of non-COVID-19 morbidity and mortality. Some authors briefly mentioned the impact of mandated school closures on child wellbeing, however, none acknowledged the unequal consequences on educational inequities and children vulnerable to experience abuse at home. This includes the effects on meeting children’s basic needs such as nutrition; studies in the UK suggested that only half of children eligible for free school meals received them during school closures in 2020 [44]. None of the papers offered detailed solutions for alternatives to school closures, or evidence to support their effectiveness. Examples could include improving classroom ventilation and use of regular testing (‘test-to-stay’) protocols. Public health policy must prioritise the most at-risk groups to protect the population as a whole [49]. Despite previous research having noted increased risk of domestic violence within unequal power relationships during the pandemic, some governments failed to enact real protection for at-risk individuals and instead simply commissioned additional research into the problem.

### Multiple inequities in COVID-19

#### Proposals

Records describing approaches to reduce multiple inequities emphasised the importance of addressing the structural causes of inequity such as racism and other social determinants of health using whole of society approaches that extend beyond the health sector (Table S6, Box S5). In Aotearoa New Zealand, a partnership among nine Iwi (Māori kin-ship groups) surveyed 18,000 constituents to identify their needs during national lockdowns [50]. In response, the partnership provided 1734 kai (food) packs, 1371 grants for home heating, 25,000 hygiene packs, and Iwi checkpoints to stop the spread of COVID-19 into Māori communities. The University College London Institute of Health Equity [51] recommended that approaches to improving the social determinants of health should be strategies that appreciate and respond to the uniqueness of communities and called for greater investment from the national government as well as the health and business sectors in cross-sector partnerships.

#### INEP commentary

There are other relevant groups not mentioned for any of the types of inequity. One prominent group is women, usually the primary carers for children and older parents. There was exacerbation of gender inequities due to lack of childcare, inability to work from home or to take time off if sick, and pressure to send children to school or day-care if sick due to the need to work. Analysis of global publicly available datasets found that between March 2020 and September 2021 women were more likely than men to report employment loss, forgoing work to care for others, and dropping out of school for reasons other than school closures [52]. Women were also more likely than men to report that gender-based violence had increased during the pandemic. Although some authors mentioned lower socio-economic groups, they did not focus specifically on workers in low-paid service industries, where the businesses either closed or the people were required go to work and be exposed (or go to work sick and expose others). Those working in higher-paid jobs had more job flexibility and the technology and internet assets to work from home. Intersectionality between gender, race, and employment in low-paid service jobs compounded inequities.

The COVID-19 pandemic has demonstrated that creating equity-based public health policy during a crisis is extremely difficult. Before the next pandemic, as well as for non-pandemic times, we need robust, evidence-based interventions to combat systemic health and social inequities to allow everyone in our communities can thrive. The Social Sector Trials, introduced in two localities in Aotearoa New Zealand, reveal how place-based initiatives have the potential to impact multiple inequities (including housing, drug and alcohol addiction, education, and training), with the objective of enhancing individual and collective self-determination [53]. These initiatives have highlighted the potential to re-orient existing program delivery to better understand the cumulative impact of services and increase shared responsibility for results [54].

## Discussion

This scoping review identified 77 publications that described approaches to specific health inequities raised or exacerbated by COVID-19 in the first year of the pandemic. Overall, the papers called for policymakers to implement equity-based pandemic response measures that acknowledge the unique needs of different communities and populations and respond accordingly. Many of the themes are echoed in the broader epidemiology and public health literature, such as work on the social determinants of health and the potential for risk prediction and other statistical models to perpetuate racism [55].

These papers provide an important starting point for developing interventions to address inequities, and the commentaries by members of the International Network for Epidemiology in Policy Equity working group identified several shortcomings. Most records were commentaries, letters, and editorials, which largely offered untested approaches that had not been implemented. We found no evaluation of the proposals and no robust evidence of their efficacy. This is in keeping with the absence of community interventions to specifically address COVID-19 listed on the US Community Preventive Services Task Force website (a collection of evidence-based community interventions, date last searched, 12 October 2023). Only a minority of suggestions focussed on equity or used a community participatory approach. We identified some suggestions that may unintentionally exacerbate health inequities—for example, approaches based on telehealth may worsen inequities for those without reliable access to internet or electronic devices. Taken together, these major gaps suggest the need for intentional governmental funding of large-scale community-based interventions which include community partners in goal setting and intervention design [43].

We identified several other limitations with the current literature. Most of the records emanated from the US and few considered across-country inequities. Along with global inequities in vaccine allocation, longer-standing health inequities among countries include access to basic public health measures, and to hospital critical care resources [56]. Data demonstrating a significantly greater burden of COVID-19 in lower income countries reflect these inequities [57]. Lack of empirical evidence of effectiveness may in part be explained by the complexity of the inequities which are not amenable to short-term resolution. For example, many younger people did not return to school at all, increasing their risk of negative health outcomes in the longer term, as education is a social determinant of health [58]. Interventions to address longer-term problems should still be tested and evaluated in the shorter term to assure there are no unintended negative consequences. Then longer-term evaluation may inform design of future measures to avoid repeating ineffective ones in the next pandemic.

Several organisations have released policy-relevant advice for addressing inequities related to COVID-19 and call for action (Supplementary Material Part 3, Box S6 and S7). Policy development is an inherently political activity [59]. Equitable policy development requires acknowledgement of systems and structures that promote inequities, as well as the worldview that these promote [49]. Paine et al. [60] underscore the importance of critiquing the world view of the researcher when claiming the results of epidemiological investigation are objective. “This claim rests on both the assumption of objectivity that data are separate from us and on the claim to represent a pre-existing, rather than socially constructed, reality” [60]. Addressing the basis of health inequities requires an investment in equitable policy development in emergency preparedness [1]. COVID-19 did not create a unique set of circumstances, instead, the pandemic served to highlight existing inequities in systems and structures. A repositioning of epidemiology to monitor the actions of policymakers shifts the focus away from individual deficits towards the “organization of society, and the role of the health system, including health policy, in creating and sustaining health inequities” [60].

There are several limitations to our review. We only included publications up to February 2021, reflected by the predominance of commentaries rather than actual research studies. Our text word search terms focussed on inequity, and this may have excluded relevant articles that used other terms (inequality or disparity). Multi-disciplinary approaches are needed for effective action to address health inequities (whether COVID related or not) but the review focusses on approaches using epidemiological methods, and the INEP group are predominantly epidemiologists.

## Conclusions

As epidemiologists, we need to reset how we study health inequities. We need to be careful in our use of methods and measurements, and question whether they propagate unscientific biases we may hold. Epidemiology must be used to address, but never to exacerbate, health inequities and inequalities.

### Supplementary Information

Below is the link to the electronic supplementary material.

Supplementary file1 (DOCX 207 KB)
