# Supplementary material for: Can evidence drive health equity in the COVID-19 pandemic and beyond?
Source: J Public Health Policy. 2024 Jan 12;45(1):137–51. doi: 10.1057/s41271-023-00452-3 (PMC10920204; doi:10.1057/s41271-023-00452-3)
Supplement: Supplementary file 1 — Supplementary file1 (DOCX 207 KB) [file 41271_2023_452_MOESM1_ESM.docx]

**Can evidence drive health equity in the COVID-19 pandemic and beyond?**

Katy Bell^a,b,*^, Sam White^b^ , Abbey Diaz,^a,c^ , Priya Bahria,^a,d^ , Fiona Sima,^a,e^ , Wael K Al-Delaimy^a,f^ , Susan dosReis^a,g^, Omar Hassan^b^ Dorothy Drabarek^b^, Monjura Nisha^b^, Kesha Baptiste-Roberts^h^, Katy Gwiazdon^a,i^, Camille Raynes-Greenow^a,b^, Robin Taylor Wilson^a,j^, James A Gaudino Jr.^a,k^ , Rafael da Silveira Moreira^a,l^ , Bruce Jennings^a,m^ , Pauline Gulliver^a,n^

**Supplementary Material**

**Table of Content:**

**Part 1. Methods**

**<add details>**

**Part 2. Results**

**<add details>**

**Part 3. Discussion**

**<add details>**

**Part 1. Methods for the Scoping Review**

**Complete search strategy for MEDLINE database**.

Database: Ovid MEDLINE® ALL <1946 to February 17, 2021>

Search Strategy:

1 Health Care Rationing/ (11749)

2 Health Equity/ (1635)

3 equit*.ti,ab. (26639)

4 inequity.ti,ab. (3676)

5 inequitable.ti,ab. (1657)

6 inequities.ti,ab. (6896)

7 1 or 2 or 3 or 4 or 5 or 6 (46404)

8 covid.ti,ab. (90869)

9 nCoV.ti,ab. (1274)

10 novel coronavirus.ti,ab. (6998)

11 new coronavirus.ti,ab. (1113)

12 SARSCoV2.ti,ab. (19)

13 SARS CoV2CoV2CoV2.ti,ab. (28624)

14 Betacoronavirus/ (33197)

15 Coronavirus Infections/ (44486)

16 8 or 9 or 10 or 11 or 12 or 13 or 14 or 15 (107464)

17 7 and 16 (1255)

**Methods for the Scoping Review**

| ***Eligibility Criteria***  Inclusion Criteria: Describe solutions that use epidemiological methods (including mixed methods) to address health inequities related to the COVID-19 pandemic; solutions are pragmatic and able to be applied in the real world. All types of publications were eligible for inclusion, including original research, commentaries, letters in peer reviewed journals or pre-print repositories.  Exclusion Criteria: Use non-epidemiological methods such as purely qualitative methods, experimental psychological studies, health economics studies; does not propose any solution, or proposed solution is not a population or health system-level solution; real-world applicability of proposed solution is unclear; does not address a health equity issue; describes a response to COVID without making recommendations.  ***Search Strategy***  We performed an initial limited search of MEDLINE and Cochrane Database of Systematic Review to identify relevant keywords and appropriate index terms with the help of an academic librarian and used these to develop the final full search strategy (Search terms used for Medline provided in Supplement 1). We searched the following databases for articles published January 1st 2020 to February 17th 2021: MEDLINE, Embase, CINAHL, the Cochrane Database of Systematic Reviews, the Cochrane COVID-19 Study Register, the COVID-evidence database, the Covid-19 trials Tracker, the Database of Publications on Coronavirus Disease (COVID-19), LitCovid, COVID-19 Open Research Dataset (CORD-19) and COVID-19 SARS-CoV-2.  ***Selection and Data Extraction***  Article selection and data extraction was performed using Covidence systematic review software (Veritas Health Innovation; Melbourne, Australia 2021). Titles and abstracts were screened by one reviewer (SW), and those that did not use epidemiological methods (broadly defined^1^) were screened out. Full-text articles were screened by two reviewers independently (SW, KB-R, or OH) according to the pre-defined eligibility criteria and disagreements were resolved by a third reviewer (KB). Charting of data from included articles was performed using a pre-defined data extraction instrument, which was developed by modifying a Joanna Briggs Institute template^1^, piloted using the first 10 included papers (SW and KB), and further refined once data extraction was underway. The data extraction template included characteristics of the data source, broad category of health inequity addressed, and the proposed solution to the inequity.  ***Data Analysis***  We first undertook descriptive analysis of the characteristics of included studies, including the broad category of health inequity being identified and addressed. We then undertook qualitative analysis within each broad health inequity category to find common themes in the proposed solutions. Finally, we critically assessed the proposed solutions to identify possible unintended consequences of solutions as well as current gaps in the evidence.   1. Peters MDJ, Godfrey C, McInerney P, Munn Z, Tricco AC, Khalil H. Chapter 11: Scoping Reviews (2020 version). In: Aromataris E, Munn Z, editors. JBI Manual for Evidence Synthesis: JBI; 2020. |
| --- |

**Figure S1 .**  **Flowchart of article screening process^2^.**


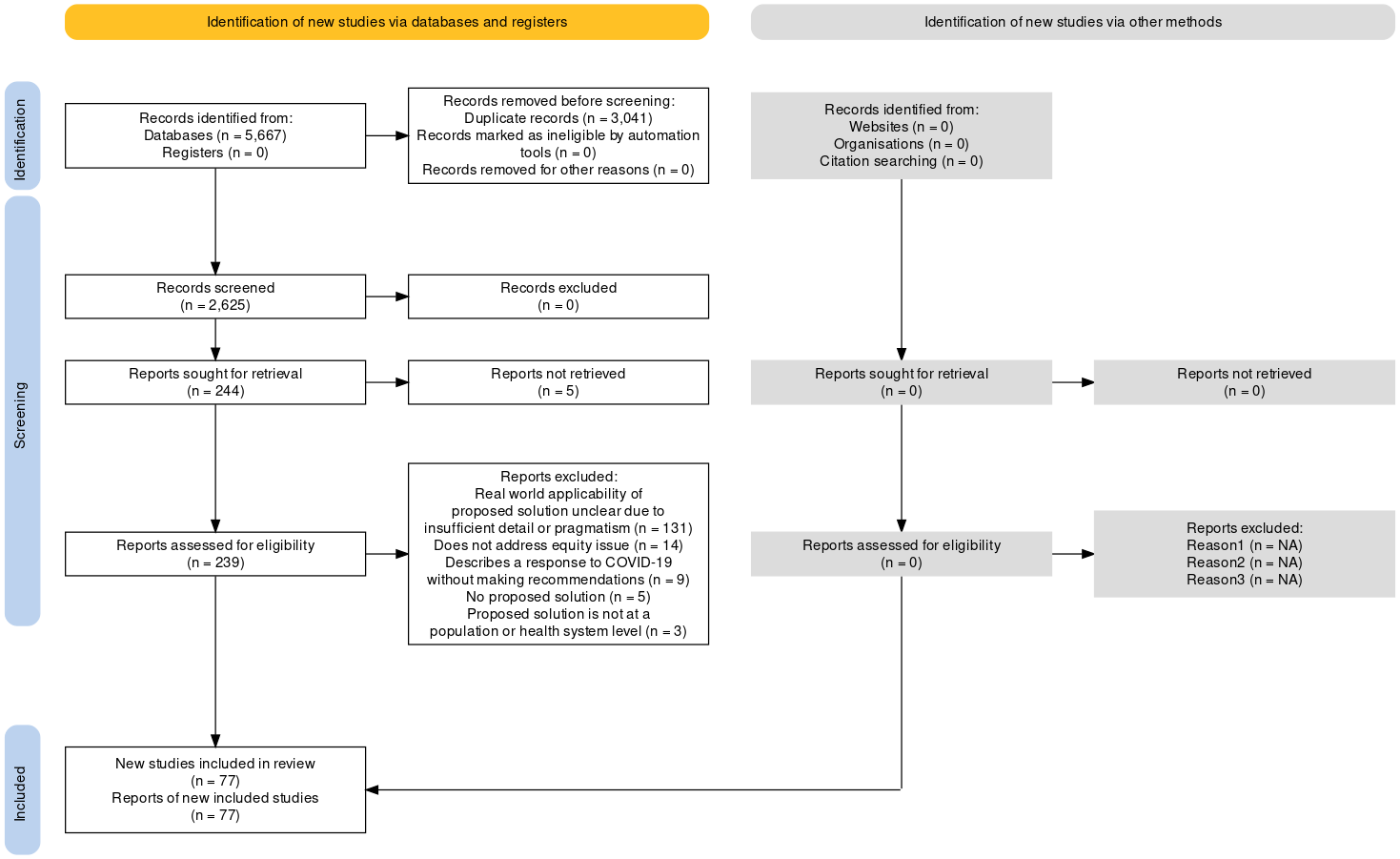
 1. Constructed using the PRISMA Flow Diagram tool. Haddaway NR, Page MJ, Pritchard CC, McGuinness LA. PRISMA2020: An R package and Shiny app for producing PRISMA 2020-compliant flow diagrams, with interactivity for optimised digital transparency and Open Synthesis. Campbell Systematic Reviews. [https://doi.org/10.1002/cl2.1230]. 2022 2022/06/01;18(2):e1230.

**Part 2. Results**

**Table S1**. Characteristics of included studies.

| Characteristic | | *n* (%) |
| --- | --- | --- |
| Location | United States^1^ | 53 (69) |
|  | Global | 17 (22) |
|  | Australia^2^ | 3 (4) |
|  | United Kingdom^2^ | 3 (4) |
|  | South Africa | 1 (1) |
|  | Aotearoa New Zealand | 1 (1) |
|  | Italy | 1 (1) |
|  | Iran | 1 (1) |
| Publication type | Commentary | 66 (86) |
|  | Letter | 3 (4) |
|  | Case report | 2 (3) |
|  | Policy review | 2 (3) |
|  | Modelling study | 1 (1) |
|  | Editorial | 1 (1) |
|  | Ethical analysis | 1 (1) |
|  | Framework description | 1 (1) |
| Setting | Population | 50 (65) |
|  | Community | 10 (13) |
|  | Acute care | 6 (8) |
|  | Prison | 1 (1) |
|  | Education system | 1 (1) |
|  | Other | 10 (13) |
| Racialised group | No specific racialised group | 55 (71) |
|  | Black American (US) | 10 (13) |
|  | Hispanic and Latinx American (US) | 8 (10) |
|  | Indigenous American peoples (US) | 2 (3) |
|  | Aboriginal and Torres Strait Islander peoples (Australia) | 1 (1) |
|  | Māori (Aotearoa New Zealand) | 1 (1) |

Notes

1. Excludes two studies which had a Global frame as well as United States

2. One study focused on United Kingdom and Australia and was counted for both

**Table S2: Approaches to inequity in risk of infection, and morbidity and mortality from, COVID-19 (n= 18)**

| **Country** | **Title** | **Author** | **Year of Publication** | **Source Type** | **Aim/Purpose** |
| --- | --- | --- | --- | --- | --- |
| Global | Social Distancing and Incarceration: Policy and Management Strategies to Reduce COVID-19 Transmission and Promote Health Equity Through Decarceration. | Henry^3^ | 2020 | Commentary | Describe how decarceration may reduce SARS-CoV-2 transmission |
| Global | Lockdowns and low- and middle-income countries: building a feasible, effective, and ethical COVID-19 response strategy. | Eyawo^4^ | 2021 | Commentary | Propose strategies low and middle income countries (LMICs) could adopt for safe and responsible lockdown entrance/exit or to avoid re-imposing coercive restrictive lockdown measures altogether |
| Global | Addressing the Syndemic of HIV, Hepatitis C, Overdose, and COVID-19 Among People Who Use Drugs: The Potential Roles for Decriminalization and Safe Supply. | Bonn^5^ | 2020 | Commentary | To propose decriminalisation and safe supply as potential solutions to addressing the syndemic between COVID and other illnesses experienced by people who use illicit drugs |
| Global | Networks of SARS-CoV-2 transmission. | Cevik^6^ | 2021 | Commentary | To propose a system for equitable resource allocation based on the size of an individual’sindividual’s social network, which reflects their relative risk of COVID exposure |
| Africa | COVID-19 Highlights the Need for Inclusive Responses to Public Health Emergencies in Africa. | Adebisi^7^ | 2021 | Commentary | To propose inclusive health strategies as solutions to address underlying health inequities in Africa that have become exacerbated during the COVID pandemic |
| Americas | COVID-19: transformative actions for more equitable, resilient, sustainable societies and health systems in the Americas. | Etienne^8^ | 2020 | Commentary | Provide recommendations for how the Americas can improve health system equity during and after the COVID pandemic |
| Canada | The COVID-19 pandemic presents an opportunity to develop more sustainable health workforces. | Bourgeault^9^ | 2020 | Commentary | Discuss attempts to increase health worker flexibility in how they respond to the pandemic, which raises the issue of under-recognised workforces. The cross-cutting issue of the need for open and flexible leadership is also highlighted. |
| Iran | Coordination, cooperation, and creativity within harm reduction networks in Iran: COVID-19 prevention and control among people who use drugs. | Alavi^10^ | 2020 | Commentary | To encourage workers from public and non-public sectors to develop innovative solutions to reduce the impact of COVID-19 on people who use drugs (PWUD) in Iran |
| UK and Australia | “Your country needs you”: the ethics of allocating staff to high-risk clinical roles in the management of patients with COVID-19. | Dunn^11^ | 2020 | Commentary | whether and how healthcare workers and their peers should be allocated to high-risk clinical roles |
| USA | Eviction, Health Inequity, and the Spread of COVID-19: Housing Policy as a Primary Pandemic Mitigation Strategy. | Benfer^12^ | 2021 | Commentary | Considers the implications of this eviction crisis for health and health inequity, and the need for eviction prevention policies during the pandemic |
| USA | COVID-19 Outbreaks at Correctional Facilities Demand a Health Equity Approach to Criminal Justice Reform. | Alohan^13^ | 2020 | Commentary | To propose criminal justice reform as a solution to the increased risk of COVID-19 infection, morbidity and mortality experienced by inmates in US correctional services facilities |
| USA | Reparations for Black American Descendants of Persons Enslaved in the U.S. and Their Estimated Impact on SARS-CoV-2 Transmission. | Richardson^14^ | 2020 | Modelling study | To model the impact of paying Black American descendants of persons enslaved in the United States reparations on the transmission of SARS-CoV-2 within the population |
| USA | Decarcerating Correctional Facilities during COVID-19: Advancing Health, Equity, and Safety | Wang^15^ | 2020 | Commentary | To discuss best practices for implementing decarceration as a strategy to mitigate the spread of COVID-19 in US correctional services facilities |
| USA | Preventing the Spread of COVID-19 in Immigration Detention Centers Requires the Release of Detainees. | Lopez^16^ | 2021 | Commentary | To advocate for the release of detainees from immigration detention centres, in order to reduce the spread of COVID-19 in this setting |
| USA | Language and Health Equity during COVID-19: Lessons and Opportunities. | Ortega^17^ | 2020 | Commentary | To develop solutions to reduce the disproportionate burden of COVID-19 on individuals with limited English proficiency |
| USA | COVID-19’s19’s Impact on the African American Community: A Stakeholder Engagement Approach to Increase Public Awareness Through Virtual Town Halls. | Fletcher^18^ | 2020 | Commentary | To reflect on the use of virtual town halls to increase public health awareness for the local African American community, and to discuss considerations for future meetings |
| USA | COVID-19 and Elder Health Inequity in Dialysis. | Wong^19^ | 2020 | Commentary | To explore the disproportionate impact of COVID-19 on ESKD patients, and develop innovative solutions |
| USA | Community Engagement of African Americans in the Era of COVID-19: Considerations, Challenges, Implications, and Recommendations for Public Health. | Henry Akintobi^20^ | 2020 | Commentary | To explore barriers to community engagement, describe response strategies by the Morehouse School of Medicine Prevention Research Centre (MSM PRC), and explore what this means for the African American community. |

**Table S3: Approaches to inequity in access to tests and to vaccines for COVID-19 (n= 13)**

| **Country** | **Title** | **Author** | **Year** | **Source Type** | **Aim/Purpose** |
| --- | --- | --- | --- | --- | --- |
| **Tests** | | | | | |
| Australia | A decentralised point-of-care testing model to address inequities in the COVID-19 response | Hengel^21^ | 2020 | Commentary | To report on a model for point-of-care COVID-19 testing amongst Aboriginal and Torres Strait Islander communities in Australia |
| USA | Spotlight on the Safety Net: Deploying Mobile COVID-19 Testing Programs in North Carolina as an Approach to Improving Health Equity. | Fiscus^22^ | 2021 | Case report | To describe several approaches to mobile COVID-19 testing implemented by North Carolina health systems, highlighting shared and divergent tactics deployed |
| **Vaccines** | | | | | |
| Global | Model-informed COVID-19 vaccine prioritization strategies by age and serostatus. | Bubar^23^ | 2021 | Commentary | Employ a model-informed approach to quantify the impact of COVID-19 vaccine prioritization strategies on cumulative incidence, mortality, and years of life lost |
| Global | Multivalue ethical framework for fair global allocation of a COVID-19 vaccine. | Liu^24^ | 2020 | Ethical analysis | Analyse four vaccine allocation paradigms and synthesise their ethical considerations to develop a model to fit the COVID-19 pandemic |
| Global | An ethical framework for global vaccine allocation. | Emanuel^25^ | 2020 | Commentary | To propose Fair Priority Model for equitable allocation of COVID-19 vaccines internationally |
| Global | Covax must go beyond proportional allocation of covid vaccines to ensure fair and equitable access. | Herzog^26^ | 2021 | Commentary | Provide critique on Covax proportional allocation model and offer alternative (Fair Priority Model) |
| Global | COVID-19 and the global public health: Tiered pricing of pharmaceutical drugs as a price-reducing policy tool | Abbas^27^ | 2020 | Commentary | To propose tiered pricing of pharmaceuticals and health technologies as a price-reducing policy option during the COVID-19 pandemic |
| Global | Equity and evidence during vaccine rollout: stepped wedge cluster randomised trials could help. | Bell^28^ | 2021 | Letter | Advocate for randomisation in vaccine roll-out, for example through stepped wedge cluster randomised trials |
| USA and Global | Framework for Equitable Allocation of COVID-19 Vaccine –– Committee on Equitable Allocation of Vaccine for the Novel Coronavirus, The National Academies of Sciences Engineering Medicine and National Academy of Medicine | Kahn^29^ | 2020 | Commentary | To develop an overarching framework for vaccine allocation to assist policy makers in the domestic and global health community. |
| USA | The Equitable Distribution of COVID-19 Therapeutics and Vaccines. | Bollyky^30^ | 2020 | Commentary | To discuss how to achieve global and equitable distribution of the COVID-19 vaccine |
| USA | What It Will Take to Equitably Distribute a COVID-19 Vaccine. | Medina-Walpole^31^ | 2021 | Commentary | Describe distribution strategy for COVID-19 vaccines developed by the American Geriatrics Society |
| USA | Transparent, equitable, safe, and effective use of COVID-19 vaccines: A societal imperative. | Abramowitz^32^ | 2020 | Editorial | Make recommendations to inform equitable allocation of SARS-CoV-2 vaccine in United States |
| USA | Ensuring Equitable Access To COVID-19 Vaccines In The US: Current System Challenges And Opportunities. | Shen^33^ | 2021 | Commentary | To inform the policy and practice of implementing equitable access to COVID-19 vaccines by providing recommendations to various stakeholders to strengthen the current immunization delivery system to ensure equitable access to all who are recommended for vaccination. |

**Table S4: Approaches to inequity in** **access to treatment for COVID-19 (n=15)**

| **Country** | **Title** | **Author** | **Year of Publication** | **Source Type** | **Aim/Purpose** |
| --- | --- | --- | --- | --- | --- |
| Global | Navigating inequities: a roadmap out of the pandemic. | Ismail^34^ | 2021 | Framework | Describe how the equity framework "Equity Matrix” may be applied to COVID-19 interventions |
| Global | COVID-19, community trials, and inclusion. | Gill^35^ | 2021 | Commentary | Describe how recruitment into platform trials to address COVID-19 can become more inclusive of communities most at risk. |
| South Africa | Age discrimination in critical care triage in South Africa: The law and the allocation of scarce health resources in the COVID-19 pandemic. | Erasmus^36^ | 2021 | Commentary | To highlight the current lack of alignment between South African ICU triage protocols and the South African constitution |
| USA | Allocating Medical Resources in the Time of Covid-19. | Brown^37^ | 2020 | Letter | To highlight the potential for discrimination against disabled, poor and minority patients during the allocation of scarce medical resourced in the pandemic response. |
| USA | Providing equitable care to patients with limited dominant language proficiency amid the COVID-19 pandemic. | Diamond^38^ | 2020 | Commentary | To develop solutions to overcome language barriers in order to combat COVID-19 healthcare disparities in LEP individuals |
| USA | Fair Allocation of Scarce Medical Resources in the Time of Covid-19. | Emanuel^39^ | 2020 | Commentary | Provide ethics-based recommendations for the fair allocation of medical resources |
| USA | Prioritizing Equity in a Time of Scarcity: The COVID-19 Pandemic. | Essien^40^ | 2020 | Commentary | To make recommendations regarding the equitable allocation of scarce medical resources. |
| USA | Fair and equitable subject selection in concurrent COVID-19 clinical trials. | Jansen^41^ | 2021 | Commentary | To develop innovative solutions for fair and equitable selection of subjects for COVID-19 trials |
| USA | Language Barriers, Equity, and COVID-19: The Impact of a Novel Spanish Language Care Group | Knuesel^42^ | 2021 | Case report | Discuss the use of a team of native Spanish-speaking doctors to assist in the clinical care of Spanish-speaking inpatients diagnosed with COVID-19 |
| USA | Allocating Medical Resources in the Time of Covid-19. Reply. | Persad^43^ | 2020 | Letter | Provide critical response to Emanuel et al’s medical resource allocation model |
| USA | Rationing, racism and justice: advancing the debate around 'colourblind' COVID-19 ventilator allocation. | Schmidt^44^ | 2021 | Policy review | Outline possible policy options towards a more just approach to rationing of healthcare resources needed for COVID-19 treatment (ventilators) than using the Sequential Organ Failure Assessment (SOFA) score which uses a single "colourblind” single creatinine threshold to estimate likelihood of death in the ICU. |
| USA | The structural and social determinants of the racial/ethnic disparities in the U.S. COVID-19 pandemic what’s our role? | Thakur^45^ | 2020 | Commentary | To provide a call to action for critical care and pulmonary specialists to advocate for more equitable patient care practises in hospitals. |
| USA | A Proposed Lottery System to Allocate Scarce COVID-19 Medications: Promoting Fairness and Generating Knowledge. | White^46^ | 2020 | Commentary | To propose lottery system to assist with equitable (random) allocation of scarce medications during COVID-19 pandemic |
| USA | Mitigating Inequities and Saving Lives with ICU Triage during the COVID-19 Pandemic. | White^47^ | 2021 | Commentary | present a practical triage framework that that incorporates these strategies and attends to the twin public health goals of promoting population health and social justice. |
| USA | Protecting Our Own: Equity for Employees as Hospitals Battle COVID-19. | Maurer^48^ | 2020 | Commentary | Provide recommendations for hospitals to address critical equity concerns among their employees |

**Table S5: Approaches to inequity in non-COVID-19 morbidity and mortality (n=8)**

| **Country** | **Title** | **Author** | **Year of Publication** | **Source Type** | **Aim/Purpose** |
| --- | --- | --- | --- | --- | --- |
| Global | A call for a gender-responsive, intersectional approach to address COVID-19. | Ryan^49^ | 2020 | Commentary | Describe critical healthcare, public health, and policy needs for implementing a gender-responsive, intersectional approach to contain the COVID-19 pandemic, mitigate the immediate and long-term consequences, and build resiliency. |
| Australia | COVID-19 pandemic: The impact on vulnerable children and young people in Australia. | Jones^50^ | 2020 | Commentary | To create awareness of disruption to usual medical care for underserved children/adolescents in Australia and to present measures that could be taken to minimise such disruption |
| UK | Considering inequalities in the school closure response to COVID-19 | Armitage^51^ | 2020 | Commentary | Address an urgent need for proactive and multifaceted responses addressing children’s social, economic, and health needs to avoid widening inequalities and honour commitments to the UN Convention on Child Rights and Sustainable Development Goals. |
| USA | Impact of COVID-19 on colorectal cancer disparities and the way forward. | Balzora^52^ | 2020 | Commentary | To explore the impact of COVID-19 on CRC screening in underserved populations, and develop plausible solutions |
| USA | Telemedicine, COVID-19, and disparities: Policy implications. | Ortega^53^ | 2020 | Policy review | Make recommendations to inform government and healthcare system policy responses to improve access to telemedicine, particularly amongst underserved patients |
| USA | Ensuring full participation of people with disabilities in an era of telehealth. | Valdez^54^ | 2021 | Commentary | To explore how transition to telehealth can increase healthcare access inequities for people with disabilities, and to explore solutions to possible challenges. |
| USA | The COVID-19 Pandemic: Identifying Adaptive Solutions for Colorectal Cancer Screening in Underserved Communities. | Nodora^55^ | 2021 | Commentary | To explore the impact of COVID-19 on CRC screening in underserved communities, and develop plausible solutions |
| USA | Setting the Agenda for Reproductive and Maternal Health in the Era of COVID-19: Lessons from a Cruel and Radical Teacher. | McCloskey^56^ | 2020 | Commentary | To create awareness of pandemic response measures disrupting access to vital and social and health resources for women and families in low-income and BIPOC communities |

**Table S6: Approaches to multiple inequities in COVID-19 (n=23)**

| **Country** | **Title** | **Author** | **Year of Publication** | **Source Type** | **Aim/Purpose** |
| --- | --- | --- | --- | --- | --- |
| Global | COVID-19 in the least developed, fragile, and conflict-affected countries - How can the most vulnerable be protected?. | Ebrahim^57^ | 2021 | Commentary | Describe a pandemic mitigation plan for least developed, fragile, or conflict-afflicted countries |
| Global | Introduction to Special Issue: Impacts of the COVID-19 Pandemic on LGBTQ+ Health and Well-Being. | Drabble^58^ | 2021 | Commentary | Interventions to inform policy and practice to better address COVID-19 in LGBTQ+ individuals and communities. |
| USA and Global | Commentary: Addressing Inequities in the Era of COVID-19: The Pandemic and the Urgent Need for Critical Race Theory | Ford^59^ | 2020 | Commentary | Describe how Public Health Critical Race Praxis (PHCRP) can be used to address inequities. |
| Aotearoa New Zealand | An Indigenous self-determination social movement response to COVID-19 | McMeeking^60^ | 2020 | Commentary | To discuss how a self-determination social movement amongst South Island Māori communities facilitated disproportionately lower infection and mortality rates compared to non-Māori. |
| Italy | CoViD-19 in Italy: homeless population needs protection | Barbieri^61^ | 2020 | Commentary | To propose operational agenda to protect homeless population based on the field experience of humanitarian group Medici per I Diritti Umani (Doctors for Human Rights, Italy) |
| UK | Addressing the national syndemic: place-based problems and solutions to UK health inequality | University College London Institute of Health Equity^62^ | 2021 | Commentary | To evaluate how effective community-based measures can be scaled up to strengthen governance and accountability for health inequity in England. |
| USA | The Color of COVID-19: Structural Racism and the Pandemic’s Disproportionate Impact on Older Racial and Ethnic Minorities | Garcia^63^ | 2021 | Commentary | To identify mechanisms through which structural racism contributes to racial inequities among older adults and make recommendations to researchers and policy-makers to help address such inequity |
| USA | Racial disparity of coronavirus disease 2019 in African American communities | Kullar^64^ | 2020 | Commentary | To explore historical reasons for health inequality in the African American community, and to develop solutions for reducing inequality in the African American community. |
| USA | Sheltering in place in a xenophobic climate: COVID-19 and children in immigrant families | Cholera^65^ | 2020 | Commentary | Assess impact of COVID-19 on Children in Immigrant Families, and advocate for potential solutions to mitigate disparities |
| USA | COVID-19 Disparities and the Black Community: A Health Equity-Informed Rapid Response Is Needed. | Brown^66^ | 2020 | Commentary | Providing strategies to combat COVID-19 |
| USA | Understanding and Addressing Latinx COVID-19 Disparities in Washington State. | Baquero^67^ | 2020 | Commentary | Describe actions taken to address inequalities and make further recommendations to address impact of the pandemic on Hispanic and Latinx communities. |
| USA | Commentary: The Invisible and Forgotten: COVID-19 Inequities Among People Experiencing Homelessness. | Iwundu^68^ | 2020 | Commentary | Describe interventions to address COVID-19 inequities among people experiencing homelessness. |
| USA | Commentary: Pandemic Inequities: Refugees' Health in the Rural United States During COVID-19. | Tippens^69^ | 2021 | Commentary | Identify strategies to improve refugees’ health and well-being related to COVID-19. |
| USA | Framing a Needed Discourse on Health Disparities and Social Inequities: Drawing Lessons from a Pandemic. | Martin-Howard^70^ | 2020 | Commentary | make recommendations for exploring factors of social equity and health inequality drawing from our experiences with COVID-19 |
| USA | Impact of COVID-19: Considerations for individuals with developmental disabilities across major life domains | Sheppard-Jones^71^ | 2021 | Commentary | Describe strategies for addressing concerns related to the impact of a pandemic on individuals with developmental disabilities |
| USA | Racism, COVID-19, and Health Inequity in the USA: a Call to Action. | Johnson-Agbakwu^72^ | 2020 | Commentary | Describe a list of concrete, actionable steps that can be taken by clinicians, public health professionals, researchers, social scientists, and policy-makers and the engagement of community stakeholders to address structural racism in advancing health equity for Black Americans. |
| USA | Using implementation science to mitigate worsening health inequities in the United States during the COVID-19 pandemic. | Jacobson^73^ | 2020 | Commentary | Explores Covid-19 responses related to: 1) testing and surveillance, 2) contact and location tracing, 3) public mask use, and 4) social distancing, as well as unintended consequences of Covid-19 policies to ensure not only an equitable pandemic response but also a more equitable society in the post-pandemic era |
| USA | Social Equity and COVID-19: The Case of African Americans. | Wright II^74^ | 2020 | Commentary | Describe strategies on how public administration scholars and practitioners can lead in crafting equitable responses to these issues to uplift African Americans during (and beyond) this global pandemic. |
| USA | COVID-19 Among African Americans: An Action Plan for Mitigating Disparities. | Peek^75^ | 2021 | Commentary | Provide recommendations for how to effectively address inequalities in the areas of data collection, COVID-19 exposure and testing, health systems collaboration, human capital repurposing, and scarce resource allocation affecting African Americans |
| USA | Advancing Equity in Public Administration: Prioritizing Equality of Outcomes in the COVID-19 Crisis. | Gadson^76^ | 2020 | Commentary | Offers practical administrative strategies to achieve social equity in pandemic response |
| USA | Addressing inequities in COVID-19 morbidity and mortality: research and policy recommendations. | Wang^77^ | 2020 | Commentary | To make research and policy recommendations regarding measures to reduce inequity in COVID-19 morbidity and mortality in the United States |
| USA | Applying the Health Justice Framework to Address Health and Health Care Inequities Experienced by People with Disabilities During and After COVID-19 | Powell^78^ | 2020 | Commentary | Provide recommendations for reducing health inequities experienced by people with disabilities both during and after the COVID pandemic |
| USA | Four COVID-19 Lessons for Achieving Health Equity. | Butler^79^ | 2020 | Commentary | Identify ways in which US health system can improve health equity using lessons learned during COVID pandemic |

**Themes and Critical Analysis of proposed solutions to inequities**

**Box S1: Proposed solutions to inequity in risk of morbidity and mortality from COVID-19 infection, onward transmission**

| **Main themes in included studies** |
| --- |
| ● Provision of free and adequate PPE, enough space to social distance, sanitation facilities and accessible healthcare including via telehealth e.g. in prisons and immigration detention centres.  ● Provide free and accessible COVID-19 testing to at risk and vulnerable communities.   ● Recognise significant risk of exposure amongst low paid workers deemed to be providing ‘essential services’ in supermarket/retail, security, janitorial and freight roles. Prioritise allocation of PPE and vaccines to these essential workers and provide paid leave to support workers being tested, vaccinated or undergoing mandatory quarantine.  ● Allocate staff for redeployment using a random lottery allocation; adopting an ""equal sharing of risk"" and a ""volunteering staff reallocation"" models for health professionals to ensure equity in high-risk clinical roles.  ● Include women, people from ethnic minorities, and people from diverse cultural and faith communities in leadership and decision-making across disciplines at the frontline.  ● Collect and release COVID-19 data by race/ethnicity and age across a range of sociodemographic characteristics.  ● Ensure linguistically and culturally appropriate public health communication with linguistic minority communities; provide free internet to make educational resources on up-to-date public health messages regarding COVID-19 accessible, and for promoting access to telemedicine services. |
| **Critical analysis of suggested solutions** |
| ● Taking an individualistic approach to prevention may not be effective when the risks are borne by communities. For example, socially distancing may not be feasible for people in overcrowded or intergenerational housing.  ● 'Telehealth' may unintentionally enlarge health inequalities by preventing access to telemedicine services for people without internet access, broadband, or a smartphone.  ● Need to take account of very substantial differences in severity of infection, rather than just differences in risk of simply being infected. The impact of the infection in terms of severity of disease and thus risk of severe morbidity and mortality, has been associated with age, racialized lived experience, socioeconomic status, underlying medical conditions, disability; all factors which are also social determinants of health. For example, there has been high morbidity and mortality from COVID-19 among people with cognitive disabilities - failures in making accessible information about the pandemic, public health measures, symptom awareness, and vaccines. Both a physical increased vulnerability among this group and societally imposed risks of poor access due to lack of effective comms and high prevalence of institutional living. |

**Box S2: Proposed** **solutions to inequity access to testing and vaccines for COVID-19**

| **Main themes in included studies** |
| --- |
| ● Develop and use equity-based allocation frameworks.  ● Include people from underserved racialized, ethnic, and sociodemographic groups in clinical trials.  ● Deliver culturally sensitive healthcare options and linguistically diverse evidence-based healthcare messaging to counter misinformation and vaccine hesitancy.  ● Locate COVID-19 testing and vaccination centres near underserved communities, in community spaces. |
| **Critical analysis of suggested solutions** |
| ● Global equity in vaccine distribution and access may be the most important solution to preventing further morbidity and mortality and deserves greater attention. Other potential solutions not considered include: the Patent waiver proposal to permit local vaccine production, use of the TRIPS agreement WTO \| around Intellectual property and pharmaceuticals to allow production of generic versions of vaccines at much cheaper cost, and learnings from evaluation of the AstraZeneca global rollout of their not-for-profit vaccine (the Oxford-AZ vaccine).  ● “Cultural sensitivity” does not ensure power sharing with communities to allow them to work alongside those who have the power to implement policy decisions. Where there is distrust in government agencies (for example, amongst poorly served populations), no amount of cultural sensitivity is going to counter that.  ● Similarly, “linguistically diverse evidence-based healthcare messaging" is important, but language is only one part of making a resource/message accessible, appropriate, relevant. This also should be co-designed with the community and designed to be health literacy sensitive. There is a need for clear, regular/frequent, consistent, honest, bipartisan supported public health/health promotion messaging.  ● Solutions need to tackle vaccine hesitancy/vaccine behaviour - especially among high risk groups, including pregnant women.  ● In places like the US without universal health coverage, access to medical treatment and vaccines can be limited by health insurance status, and out-of-pocket costs. Making medications, vaccines, and tests low cost is needed for access and utilization. |

**Box S3: Main themes in proposed solutions to inequity in access to treatment for COVID-19**

| **Main themes in included studies** |
| --- |
| ● Provide culturally tailored/sensitive COVID-19 information, support and medical care.  ● Include people from underserved racialized, ethnic, and sociodemographic groups in clinical trials.  ● Increase diversity in institutional triage committees and revise critical care triage guidelines to prevent discrimination of underserved groups; apply equity weighting for structural inequities; do not allocate based on longer-term survival. Prioritise front line and essential workers for treatment. |
| **Critical analysis of suggested solutions** |
| ● Equity based allocation frameworks need to also protect against discrimination based on age. Leaving it up to physicians to decide who would be offered ICU and other treatment based on success of survival disadvantaged older patients, as well as those from racialized groups. The value of human life should not be based on future productivity. We value elders for their wisdom and what they have provided for the community. Algorithms of risk, first come first serve, or pure clinically based decision can disadvantage the most vulnerable in society.  ● Including under-served populations in clinical trials is insufficient, communities need to help co-design clinical trials, including recruitment strategies that are appropriate, safe, and accessible. |

**Box S4: Main themes in proposed solutions to inequity in** **non-COVID-19 morbidity and mortality during the pandemic**

|  |
| --- |
| ● User-friendly telehealth technologies, increasing funding in order to make high speed internet available in underserved communities.  ● Increase community doctors available to perform procedures in underserved communities and involve support workers and family members in care  ● Ensure data collected reflects community diversity and use multiple methodologies, including mixed methods research, to capture lived experiences. Conduct research focused on children and their experience during the pandemic, women’s experiences, domestic violence, maternal health, and mental health.  ● Implement cross-sectoral interventions including fair pay, paid leave and sick days, paid family leave, baby bonds, and supplemental income.  ● Implement alternative COVID-19 risk mitigation strategies to school closures and prioritise usual medical care for children during the pandemic.  ● Community-based partnerships to ensure most pressing needs are responded to. |
| **Critical analysis of suggested solutions** |
| ● 'Telehealth' may unintentionally enlarge health inequalities by preventing access to telemedicine services for people without internet access, broadband, or a smartphone.  ● Detailed solutions to keeping schools open not provided, but might include improving air quality and good ventilation, and the test to stay strategy (latter has RCT evidence to support).  ● Violence within unequal power relationships during the pandemic was expected and while conducting research into experiences of this may provide confirmatory evidence, it is not an actual solution. What was needed early on was for governments to heed the concerns of advocacy groups with respect to the lock down measures and subsequent “vaccine control” by violent partners. A wider issue is that while some governments followed public health advice, there was a reluctance to prioritise some groups over others, despite the existence of previous research that told us a pandemic would exacerbate current inequities. Policy needs to focus on those most at risk in order to protect the population as a whole. |

**Box S5: Main themes in proposed solutions to multiple inequities in COVID-19**

| **Main themes in included studies** |
| --- |
| ● Collaboration between community-based organisations and healthcare providers to ensure: culturally appropriate health services and public health messaging; support provision of and adherence to COVID control measures, and social support including affordable/free child care, alternative housing, paid leave.   ● Conduct community-engaged and participatory research and evaluation.   ● Identify and address root causes of health inequity such as structural racism and the social determinants of health  ● Create standardized data collection systems that captures racial/ethnic data with COVID-19 reporting  ● Explicitly target racism and its determinants in public health interventions  ● Increase diversity of healthcare workers to better represent the ethno-cultural and linguistic diversity of the local communities served.  ● Employ whole of government approach to improving the social determinants of health through place-based strategies coordinated by local governments/community organisations that are based on a willingness to appreciate and respond to the uniqueness of different communities. |
| **Critical analysis of suggested solutions** |
| ● There are other examples of successful community led interventions not described in the included papers  ●Need to also consider impact on inequities when pre-pandemic planning. It is difficult to address these in the context of a crisis, they require attention before and after the pandemic has occurred.  ● Much of the increased risk for older people was associated with their place of residence - institutional care AND multi-generational households: much increased risk over age alone. |

**Part 3. Discussion**

**Box S6: Policy Recommendations to address health equity concerns related to COVID-19**

| • The World Health Organization Regional Office statement^1^ provided strategies that were aligned with the United Nations socioeconomic response pillars^2^, including calls for equitable and sustainable access to health care and essential services such as education, heating, sanitation and internet access.  • The Organisation for Economic Co-operation and Development (OECD) statement^3^ advocates for differentiated region-based pandemic responses within countries to meet local needs and avoid unnecessary impacts from nation-wide lockdowns. The OECD calls for COVID-19 data to be stratified by region and for greater information sharing and cooperation between national and subnational governments.  • The American Public Health Association’s comprehensive set of recommendations^4^ include: coordinating and funding a universal public health system, comprehensive collection of sociodemographic data for case reporting, universal access to healthcare and mental healthcare, housing, food, health insurance, health literacy education, broadband internet, access to substance use treatment, and school lunch programs, a need to engage with and empower communities in ways to enable improved access and uptake to vaccines, testing, healthcare, and education, and development of workplace safety and health standards, including universal paid sick leave, to help reduce disease spread and illness that occurs in the workplace.  • The American College of Obstetricians and Gynaecologists statement^5^ focuses on exacerbation of racial disparities in maternal morbidity and mortality in the US during the pandemic and calls for COVID-19 testing and outcome data to be collected at local, state-wide and national levels and stratified by race, socioeconomic status and predominant language. Clinicians are encouraged to screen for, and document, social factors that may compromise an individual’s health or access to care, as well as identify and appropriately refer patients with evidence of psychological distress during the pandemic.  • The US Centres for Disease Control and Prevention statement^6^ outlines strategies to improve vaccine equity within the US population, including tailoring strategies to address misinformation and vaccine hesitancy through culturally relevant messaging delivered in the predominant languages spoken within specific communities. There was also a call to employ more vaccination workers that shared similar racialized, cultural, and religious characteristics with the communities in which they were working.  1. World Health Organisation (WHO). Health inequity and the effects of COVID-19: accessing, responding to and mitigating the socioeconomic imkpact on health to build a better future. Copenhagen; 2020. [<https://apps.who.int/iris/handle/10665/338199> ].  2. United Nations (UN). A UN framework for the immediate socio-economic response to COVID‑19. New York, USA; 2020. [<https://unsdg.un.org/resources/un-framework-immediate-socio-economic-response-covid-19> ].  3. Organisation for Economic Co-operation and Development (OECD). The territorial impact of COVID-19: Managing the crisis and recovery across levels of government. Paris, France; 2021. [<https://www.oecd.org/coronavirus/policy-responses/the-territorial-impact-of-covid-19-managing-the-crisis-and-recovery-across-levels-of-government-a2c6abaf/> ].  4. American Public Health Association. Call for Urgent Actions to Address Health Inequities in the U.S. Coronavirus Disease 2019 Pandemic and Response. USA; 2021. [<https://www.apha.org/policies-and-advocacy/public-health-policy-statements/policy-database/2021/01/13/health-inequities-in-the-us-coronavirus-disease-2019-pandemic-and-response>]  5. American College of Obstetricians and Gynecologists. Addressing health equity during the COVID-19 pandemic. Washington, DC; 2020. [<https://www.acog.org/clinical-information/policy-and-position-statements/position-statements/2020/addressing-health-equity-during-the-covid-19-pandemic> ].  6. Centre for Disease Control (CDC). Health equity considerations and racial and ethnic minority groups. Atlanta, GA; 2022. [<https://www.cdc.gov/coronavirus/2019-ncov/community/health-equity/race-ethnicity.html>]. |
| --- |

**Box S7: Call to Action for Health Policy**

| The International Network of Epidemiology in Policy (<https://epidemiologyinpolicy.org/> ) is as an organization that promotes integrity, equity, and evidence in policies impacting health to better protect the health of the whole community^1^. We call on health policy makers to take the following actions to prevent and mitigate inequities threatening health and well-being of our communities from COVID-19 and other diseases:  1. Urgently address inequity between countries in access to vaccines and curative treatments.  2. Co-create, co-design, and co-produce equity-based interventions with communities, focusing on those most at risk to protect them as well as the population as a whole.  3. Epidemiologists may play a key role in this, in collaboration with people from other relevant disciplines, to develop effective systems-based methods and approaches to improving health equity.  4. Test potential solutions and undertake robust evaluation to determine feasibility, identify potential adverse effects, and ensure that potential benefits outweigh potential harms (including over the longer term).  1. Oremus M, Taylor-Wilson R, Aldrich M, et al. The role of epidemiologists in SARS-CoV-2 and COVID-19 research. Public Health. 2021;190:e3-e4. |
| --- |

**References**

1. Smith MY, Hogan SA, Jack SM, Wilson RT, Oremus M. The case for using mixed methods for designing, implementing, and disseminating evidence-based interventions for public health practice. J Public Health Policy. 2022 Jun;43(2):292-303.

2. Haddaway NR, Page MJ, Pritchard CC, McGuinness LA. PRISMA2020: An R package and Shiny app for producing PRISMA 2020-compliant flow diagrams, with interactivity for optimised digital transparency and Open Synthesis. Campbell Systematic Reviews. [<https://doi.org/10.1002/cl2.1230>]. 2022 2022/06/01;18(2):e1230.

3. Henry BF. Social Distancing and Incarceration: Policy and Management Strategies to Reduce COVID-19 Transmission and Promote Health Equity Through Decarceration. Health education & behavior : the official publication of the Society for Public Health Education. 2020;47(4):536-9.

4. Eyawo O, Viens AM, Ugoji UC. Lockdowns and low- and middle-income countries: building a feasible, effective, and ethical COVID-19 response strategy. Globalization and health. 2021;17(1):13.

5. Bonn M, Palayew A, Bartlett S, Brothers TD, Touesnard N, Tyndall M. Addressing the Syndemic of HIV, Hepatitis C, Overdose, and COVID-19 Among People Who Use Drugs: The Potential Roles for Decriminalization and Safe Supply. Journal of studies on alcohol and drugs. 2020;81(5):556-60.

6. Cevik M, Baral SD. Networks of SARS-CoV-2 transmission. Science. 2021;373(6551):162-3.

7. Adebisi YA, Ekpenyong A, Ntacyabukura B, Lowe M, Jimoh ND, Abdulkareem TO, et al. COVID-19 Highlights the Need for Inclusive Responses to Public Health Emergencies in Africa. The American journal of tropical medicine and hygiene. 2020(3zq, 0370507).

8. Etienne CF, Fitzgerald J, Almeida G, Birmingham ME, Brana M, Bascolo E, et al. COVID-19: transformative actions for more equitable, resilient, sustainable societies and health systems in the Americas. BMJ global health. 2020;5(8).

9. Bourgeault IL, Maier CB, Dieleman M, Ball J, MacKenzie A, Nancarrow S, et al. The COVID-19 pandemic presents an opportunity to develop more sustainable health workforces. Human resources for health. 2020;18(1):83.

10. Alavi M, Moghanibashi-Mansourieh A, Radfar SR, Alizadeh S, Bahramabadian F, Esmizade S, et al. Coordination, cooperation, and creativity within harm reduction networks in Iran: COVID-19 prevention and control among people who use drugs. The International journal on drug policy. 2020(9014759):102908.

11. Dunn M, Sheehan M, Hordern J, Turnham HL, Wilkinson D. 'Your country needs you': the ethics of allocating staff to high-risk clinical roles in the management of patients with COVID-19. J Med Ethics. 2020;46(7):436-40.

12. Benfer EA, Vlahov D, Long MY, Walker-Wells E, Pottenger JL, Jr., Gonsalves G, et al. Eviction, Health Inequity, and the Spread of COVID-19: Housing Policy as a Primary Pandemic Mitigation Strategy. Journal of urban health : bulletin of the New York Academy of Medicine. 2021;98(1):1-12.

13. Alohan D, Calvo M. COVID-19 Outbreaks at Correctional Facilities Demand a Health Equity Approach to Criminal Justice Reform. Journal of urban health : bulletin of the New York Academy of Medicine. 2020;97(3):342-7.

14. Richardson ET, Malik MM, Darity WA, Mullen AK, Malik M, Benton A, et al. Reparations for Black American Descendants of Persons Enslaved in the U.S. and Their Estimated Impact on SARS-CoV-2 Transmission. medRxiv : the preprint server for health sciences. 2020(101767986).

15. National Academies of Sciences E, Medicine, Division of B, Social S, Education, Committee on L, et al. Decarcerating Correctional Facilities during COVID-19: Advancing Health, Equity, and Safety. Schuck J, Backes EP, Western B, Wang EA, editors. Washington (DC): National Academies Press (US)

Copyright 2020 by the National Academy of Sciences. All rights reserved.; 2020.

16. Lopez WD, Kline N, LeBron AMW, Novak NL, De Trinidad Young M-E, Gonsalves G, et al. Preventing the Spread of COVID-19 in Immigration Detention Centers Requires the Release of Detainees. American journal of public health. 2021;111(1):110-5.

17. Ortega P, Martinez G, Diamond L. Language and Health Equity during COVID-19: Lessons and Opportunities. Journal of health care for the poor and underserved. 2020;31(4):1530-5.

18. Fletcher FE, Allen S, Vickers SM, Beavers T, Hamlin CM, Young-Foster D, et al. COVID-19's Impact on the African American Community: A Stakeholder Engagement Approach to Increase Public Awareness Through Virtual Town Halls. Health equity. 2020;4(1):320-5.

19. Wong LP. COVID-19 and Elder Health Inequity in Dialysis. Kidney medicine. 2020;2(6):675-7.

20. Henry Akintobi T, Jacobs T, Sabbs D, Holden K, Braithwaite R, Johnson LN, et al. Community Engagement of African Americans in the Era of COVID-19: Considerations, Challenges, Implications, and Recommendations for Public Health. Preventing chronic disease. 2020;17(101205018):E83.

21. Hengel B, Causer L, Matthews S, Smith K, Andrewartha K, Badman S, et al. A decentralised point-of-care testing model to address inequities in the COVID-19 response. The Lancet Infectious Diseases. 2021((Hengel, Causer, Smith, Badman, Tangey, Cunningham, Watts, King, Applegate, Guy) Kirby Institute, University of New South Wales, Sydney, NSW, Australia(Matthews, Andrewartha, Spaeth, Phillips, Shephard) International Centre for Point-of-Care Testing, Flin).

22. Fiscus L, Towns R, Wood S, Oliver P, Fox S, Weathers A, et al. Spotlight on the Safety Net: Deploying Mobile COVID-19 Testing Programs in North Carolina as an Approach to Improving Health Equity. North Carolina medical journal. 2021;82(1):80-2.

23. Bubar KM, Reinholt K, Kissler SM, Lipsitch M, Cobey S, Grad YH, et al. Model-informed COVID-19 vaccine prioritization strategies by age and serostatus. medRxiv : the preprint server for health sciences. 2020(101767986).

24. Liu Y, Salwi S, Drolet BC. Multivalue ethical framework for fair global allocation of a COVID-19 vaccine. Journal of medical ethics. 2020;46(8):499-501.

25. Emanuel EJ, Persad G, Kern A, Buchanan A, Fabre C, Halliday D, et al. An ethical framework for global vaccine allocation. Science (New York, NY). 2020;369(6509):1309-12.

26. Herzog LM, Norheim OF, Emanuel EJ, McCoy MS. Covax must go beyond proportional allocation of covid vaccines to ensure fair and equitable access. BMJ (Clinical research ed). 2021;372(8900488, bmj, 101090866):m4853.

27. Abbas MZ. COVID-19 and the global public health: Tiered pricing of pharmaceutical drugs as a price-reducing policy tool. Journal of Generic Medicines. 2020((Abbas) Faculty of Law, Queensland University of Technology, Brisbane, Australia).

28. Bell KJL, Glasziou P, Stanaway F, Bossuyt P, Irwig L. Equity and evidence during vaccine rollout: stepped wedge cluster randomised trials could help. BMJ. 2021.

29. National Academies of Sciences E, Medicine, Health, Medicine D, Board on Population H, Public Health P, et al. Framework for Equitable Allocation of COVID-19 Vaccine. Kahn B, Brown L, Foege W, Gayle H, editors. Washington (DC): National Academies Press (US)

Copyright 2020 by the National Academy of Sciences. All rights reserved.; 2020.

30. Bollyky TJ, Gostin LO, Hamburg MA. The Equitable Distribution of COVID-19 Therapeutics and Vaccines. JAMA. 2020;323(24):2462-3.

31. Medina-Walpole A. What it will take to equitably distribute a COVID-19 vaccine. Geriatric nursing (New York, NY). 2020;41(6):1028-9.

32. Abramowitz PW, Cobaugh DJ, Thompson KK. Transparent, equitable, safe, and effective use of COVID-19 vaccines: A societal imperative. American journal of health-system pharmacy : AJHP : official journal of the American Society of Health-System Pharmacists. 2020;77(24):2021-2.

33. Shen AK, Hughes Iv R, DeWald E, Rosenbaum S, Pisani A, Orenstein W. Ensuring Equitable Access To COVID-19 Vaccines In The US: Current System Challenges And Opportunities. Health affairs (Project Hope). 2021;40(1):62-9.

34. Ismail SJ, Tunis MC, Zhao L, Quach C. Navigating inequities: a roadmap out of the pandemic. BMJ global health. 2021;6(1).

35. Gill PS, Poduval S, Thakur JS, Iqbal R. COVID-19, community trials, and inclusion. Lancet. 2021;397(10279):1036-7.

36. Erasmus N. Age discrimination in critical care triage in South Africa: The law and the allocation of scarce health resources in the COVID-19 pandemic. South African medical journal = Suid-Afrikaanse tydskrif vir geneeskunde. 2020;110(12):1172-5.

37. Brown MJ, Goodwin J. Allocating Medical Resources in the Time of Covid-19. The New England journal of medicine. 2020;382(22):e79.

38. Diamond LC, Jacobs EA, Karliner L. Providing equitable care to patients with limited dominant language proficiency amid the COVID-19 pandemic. Patient education and counseling. 2020;103(8):1451-2.

39. Emanuel EJ, Persad G, Upshur R, Thome B, Parker M, Glickman A, et al. Fair Allocation of Scarce Medical Resources in the Time of Covid-19. The New England journal of medicine. 2020;382(21):2049-55.

40. Essien UR, Eneanya ND, Crews DC. Prioritizing Equity in a Time of Scarcity: The COVID-19 Pandemic. Journal of general internal medicine. 2020;35(9):2760-2.

41. Jansen MO, Angelos P, Schrantz SJ, Donington JS, Madariaga MLL, Zakrison TL. Fair and equitable subject selection in concurrent COVID-19 clinical trials. Journal of medical ethics. 2021;47(1):7-11.

42. Knuesel S, Chuang W, Olson E, Betancourt J. Language Barriers, Equity, and COVID-19: The Impact of a Novel Spanish Language Care Group. Journal of hospital medicine. 2020((Knuesel, Chuang, Betancourt) Division of General Internal Medicine, Department of Medicine, Massachusetts General Hospital, Boston, MA(Olson, Betancourt) Center for Diversity & Inclusion, Massachusetts General Hospital, Boston, MA).

43. Persad G, Phillips J, Emanuel EJ. Allocating Medical Resources in the Time of Covid-19. Reply. The New England journal of medicine. 2020;382(22):e79.

44. Schmidt H, Roberts DE, Eneanya ND. Rationing, racism and justice: advancing the debate around 'colourblind' COVID-19 ventilator allocation. Journal of medical ethics. 2021(j1d, 7513619).

45. Thakur N, Lovinsky-Desir S, Bime C, Wisnivesky JP, Celedon JC. The structural and social determinants of the racial/ethnic disparities in the U.S. COVID-19 pandemic what's our role? American Journal of Respiratory and Critical Care Medicine. 2020;202(7):943-9.

46. White DB, Angus DC. A Proposed Lottery System to Allocate Scarce COVID-19 Medications: Promoting Fairness and Generating Knowledge. JAMA. 2020;324(4):329-30.

47. White DB, Lo B. Mitigating Inequities and Saving Lives with ICU Triage during the COVID-19 Pandemic. American journal of respiratory and critical care medicine. 2021;203(3):287-95.

48. Maurer LR, Perez NP, Witt EE, Ortega G. Protecting Our Own: Equity for Employees as Hospitals Battle COVID-19. Health equity. 2020;4(1):394-6.

49. Ryan NE, El Ayadi AM. A call for a gender-responsive, intersectional approach to address COVID-19. Global public health. 2020;15(9):1404-12.

50. Jones B, Woolfenden S, Pengilly S, Breen C, Cohn R, Biviano L, et al. COVID-19 pandemic: The impact on vulnerable children and young people in Australia. Journal of paediatrics and child health. 2020;56(12):1851-5.

51. Armitage R, Nellums LB. Considering inequalities in the school closure response to COVID-19. Lancet Glob Health. 2020.

52. Balzora S, Issaka RB, Anyane-Yeboa A, Gray DM, 2nd, May FP. Impact of COVID-19 on colorectal cancer disparities and the way forward. Gastrointestinal endoscopy. 2020;92(4):946-50.

53. Ortega G, Rodriguez JA, Maurer LR, Witt EE, Perez N, Reich A, et al. Telemedicine, COVID-19, and disparities: Policy implications. Health policy and technology. 2020;9(3):368-71.

54. Valdez RS, Rogers CC, Claypool H, Trieshmann L, Frye O, Wellbeloved-Stone C, et al. Ensuring full participation of people with disabilities in an era of telehealth. Journal of the American Medical Informatics Association : JAMIA. 2021;28(2):389-92.

55. Nodora JN, Gupta S, Howard N, Motadel K, Propst T, Rodriguez J, et al. The COVID-19 Pandemic: Identifying Adaptive Solutions for Colorectal Cancer Screening in Underserved Communities. Journal of the National Cancer Institute. 2020(j9j, 7503089).

56. McCloskey L, Amutah-Onukagha N, Bernstein J, Handler A. Setting the Agenda for Reproductive and Maternal Health in the Era of COVID-19: Lessons from a Cruel and Radical Teacher. Maternal and child health journal. 2021;25(2):181-91.

57. Ebrahim SH, Gozzer E, Ahmed Y, Imtiaz R, Ditekemena J, Rahman NMM, et al. COVID-19 in the least developed, fragile, and conflict-affected countries - How can the most vulnerable be protected? International journal of infectious diseases : IJID : official publication of the International Society for Infectious Diseases. 2021;102(c3r, 9610933):381-8.

58. Drabble LA, Eliason MJ. Introduction to Special Issue: Impacts of the COVID-19 Pandemic on LGBTQ+ Health and Well-Being. Journal of homosexuality. 2021;68(4):545-59.

59. Ford CL. Commentary: Addressing Inequities in the Era of COVID-19: The Pandemic and the Urgent Need for Critical Race Theory. Family & community health. 2020;43(3):184-6.

60. McMeeking S, Leahy H, Savage C. An Indigenous self-determination social movement response to COVID-19. AlterNative. 2020;16(4):395-8.

61. Barbieri A. Covid-19 in Italy: Homeless population needs protection. Recenti Progressi in Medicina. 2020;111(5):E1-E2.

62. Equity UCLIoH. Addressing the national syndemic: place-based problems and solutions to UK health inequality: University College London Institute of Health Equity2021.

63. Garcia MA, Homan PA, Garcia C, Brown TH. The Color of COVID-19: Structural Racism and the Pandemic's Disproportionate Impact on Older Racial and Ethnic Minorities. The journals of gerontology Series B, Psychological sciences and social sciences. 2020((Garcia) University of Nebraska Sociology & Institute of Ethnic Studies Oldfather Hall Lincoln, NE, Italy(Homan) Florida State University Department of Sociology Pepper Institute on Aging and Public Policy(Garcia) University of Nebraska Department of Soci).

64. Kullar R, Marcelin JR, Swartz TH, Piggott DA, Macias Gil R, Mathew TA, et al. Racial Disparity of Coronavirus Disease 2019 in African American Communities. The Journal of infectious diseases. 2020;222(6):890-3.

65. Cholera R, Falusi OO, Linton JM. Sheltering in place in a xenophobic climate: COVID-19 and children in immigrant families. Pediatrics. 2020;146(1):e20201094.

66. Brown IM, Khan A, Slocum J, Campbell LF, Lacey JR, Landry AM. COVID-19 Disparities and the Black Community: A Health Equity-Informed Rapid Response Is Needed. American journal of public health. 2020;110(9):1350-1.

67. Baquero B, Gonzalez C, Ramirez M, Chavez Santos E, Ornelas IJ. Understanding and Addressing Latinx COVID-19 Disparities in Washington State. Health education & behavior : the official publication of the Society for Public Health Education. 2020;47(6):845-9.

68. Iwundu CN, Santa Maria D, Hernandez DC. Commentary: The Invisible and Forgotten: COVID-19 Inequities Among People Experiencing Homelessness. Family & community health. 2021;44(2):108-9.

69. Tippens JA, Springer PR. Commentary: Pandemic Inequities: Refugees' Health in the Rural United States During COVID-19. Family & community health. 2021;44(2):102-7.

70. Martin-Howard S, Kyle Farmbry JD. Framing a Needed Discourse on Health Disparities and Social Inequities: Drawing Lessons from a Pandemic. Public administration review. 2020(qin, 0045715).

71. Sheppard-Jones K, Avellone L, Rumrill P, Seward H. Impact of COVID-19: Considerations for individuals with developmental disabilities across major life domains. Journal of Vocational Rehabilitation. 2021;54(1):5-13.

72. Johnson-Agbakwu CE, Ali NS, Oxford CM, Wingo S, Manin E, Coonrod DV. Racism, COVID-19, and Health Inequity in the USA: a Call to Action. Journal of racial and ethnic health disparities. 2020(101628476).

73. Jacobson TA, Smith LE, Hirschhorn LR, Huffman MD. Using implementation science to mitigate worsening health inequities in the United States during the COVID-19 pandemic. International journal for equity in health. 2020;19(1):170.

74. Wright JE, 2nd, Merritt CC. Social Equity and COVID-19: The Case of African Americans. Public administration review. 2020(qin, 0045715).

75. Peek ME, Simons RA, Parker WF, Ansell DA, Rogers SO, Edmonds BT. COVID-19 Among African Americans: An Action Plan for Mitigating Disparities. American journal of public health. 2021;111(2):286-92.

76. Gadson DN. Advancing Equity in Public Administration: Prioritizing Equality of Outcomes in the COVID-19 Crisis. Risk, hazards & crisis in public policy. 2020(101734657).

77. Wang ML, Behrman P, Dulin A, Baskin ML, Buscemi J, Alcaraz KI, et al. Addressing inequities in COVID-19 morbidity and mortality: research and policy recommendations. Translational behavioral medicine. 2020;10(3):516-9.

78. Powell R. Applying the Health Justice Framework to Address Health and Health Care Inequities Experienced by People with Disabilities During and After COVID-19. 2020.

79. Butler SM. Four COVID-19 Lessons for Achieving Health Equity. JAMA. 2020;324(22):2245-6.
